# E46K-like α-synuclein mutants increase lipid interactions and disrupt membrane selectivity

**DOI:** 10.1074/jbc.RA118.006551

**Published:** 2019-05-02

**Authors:** Matteo Rovere, Alex E. Powers, Haiyang Jiang, Julia C. Pitino, Luis Fonseca-Ornelas, Dushyant S. Patel, Alessandro Achille, Ralf Langen, Jobin Varkey, Tim Bartels

**Affiliations:** From the ‡ Ann Romney Center for Neurologic Diseases, Brigham and Women's Hospital and Harvard Medical School, Boston, Massachusetts 02115,; the § Department of Computer Science, University of California, Los Angeles, California 90095,; the ¶ Zilkha Neurogenetic Institute, University of Southern California, Los Angeles, California 90033, and; the ‖ Dementia Research Institute, University College London, London WC1E 6BT, United Kingdom

**Keywords:** α-synuclein, Parkinson disease, neurodegeneration, intrinsically disordered protein, protein–lipid interaction, CD, isothermal titration calorimetry (ITC), 11/3 helix, large unilamellar vesicle, small unilamellar vesicle

## Abstract

Parkinson's disease (PD) is one of the most common neurodegenerative disorders, and both genetic and histopathological evidence have implicated the ubiquitous presynaptic protein α-synuclein (αSyn) in its pathogenesis. Recent work has investigated how disrupting αSyn's interaction with membranes triggers trafficking defects, cellular stress, and apoptosis. Special interest has been devoted to a series of mutants exacerbating the effects of the E46K mutation (associated with autosomal dominant PD) through homologous Glu-to-Lys substitutions in αSyn's N-terminal region (*i.e.* E35K and E61K). Such E46K-like mutants have been shown to cause dopaminergic neuron loss and severe but L-DOPA–responsive motor defects in mouse overexpression models, presenting enormous translational potential for PD and other “synucleinopathies.” In this work, using a variety of biophysical techniques, we characterize the molecular pathology of E46K-like αSyn mutants by studying their structure and membrane-binding and remodeling abilities. We find that, although a slight increase in the mutants' avidity for synaptic vesicle–like membranes can be detected, most of their deleterious effects are connected to their complete disruption of αSyn's curvature selectivity. Indiscriminate binding can shift αSyn's subcellular localization away from its physiological interactants at the synaptic bouton toward trafficking vesicles and organelles, as observed in E46K-like cellular and murine models, as well as in human pathology. In conclusion, our findings suggest that a loss of curvature selectivity, rather than increased membrane affinity, could be the critical dyshomeostasis in synucleinopathies.

## Introduction

Parkinson's disease (PD)^3^ is a progressive neurodegenerative disorder characterized in its early stages by resting tremor, bradykinesia, and rigidity because of loss of dopaminergic neurons in the substantia nigra (1). Its prevalence in industrialized countries is estimated at 1% in people over 60 years of age, making it the second most common neurodegenerative disorder after Alzheimer's disease (2). α-Synuclein (αSyn), a small presynaptic protein ubiquitously expressed in nervous tissue (3), has long been considered a key player in the pathogenesis of PD, as supported by both genetic and histopathologic evidence (4). Its role in neuronal physiology, although still debated, has been tied to the maintenance of synaptic homeostasis (5, 6). These mechanisms are especially important in dopaminergic neurons, which exhibit high-frequency activity bursts (7).

One of the recurring themes of αSyn biology is its affinity for lipid interfaces and how such interplay shapes its function and dysfunction. Although αSyn has been classically considered an intrinsically disordered protein (8, 9), its N-terminal region contains seven imperfect 11-mer repeats (with the consensus *X*KTKEGV*XXXX*), homologous to those of apolipoproteins (10–12). In fact, upon interaction with liposomes and micelles, αSyn undergoes a coil-to-helix transition and binds robustly to curved, negatively charged interfaces (13, 14). In particular, its specificity for lipid bilayers similar in charge and curvature to those of synaptic vesicles (15) is attained by virtue of the periodic arrangement of mildly hydrophobic and hydrophilic amino acid side chains (16). In addition, the numerous lysines in the N-terminal region mediate the formation of ionic bridges with the charged heads of negative membranes (17). αSyn's N terminus has also been shown to be critical for its subcellular localization to the presynaptic terminals (18) and its partitioning between the cytosol and membranes, regulated by electrical activity (19).

Along with its role in cellular physiology, the lipid-binding ability of αSyn's N-terminal region has also been implicated in the pathogenesis of neurodegeneration (20). Overexpression of αSyn and its mutants associated with autosomal dominant PD (fPD) in yeast and mammalian cell models severely compromises vesicle trafficking and protein clearance through abnormal membrane association (21–23). In addition, vesicle and organelle clusters have been recognized as prominent features of Lewy pathology (24).

Recent work has shown that modifications in αSyn's repeats that enhance the amphipathic nature of its membrane-binding helix modulate toxicity upon overexpression (25, 26). Inserting either more hydrophobic residues on the largely apolar face of the helix or charged residues on the cytosol-exposed side appears to increase the ability of αSyn mutants to induce toxicity and cluster a variety of intracellular vesicles and organelles into αSyn-rich inclusions. Among these mutants, Glu-to-Lys mutations homologous to the fPD-associated E46K mutation (27) (*e.g.* E35K and E61K) are of particular interest because of their direct connection to disease and their robust toxicity in cellular models (28). Readouts of cellular toxicity (*e.g.* viability and apoptotic markers) and their ability to cluster vesicles correlate with the number of Glu-to-Lys substitutions, suggesting a common molecular mechanism between the E46K mutation and these “exacerbated” E46K-like mutants. In addition, such mutants have been shown to markedly decrease the ratio between aggregation-resistant αSyn tetramers (29) and aggregation-prone monomers, whose imbalance has been proposed as one of the molecular mechanisms underlying disease inception (28, 30). Lastly, heterozygous transgenic mice overexpressing E46K-like αSyn mutants present a robust and progressive PD-like phenotype with severe tremor and gait deficits (partially rescued by L-DOPA administration), loss of dopaminergic neurons, and formation of protease-resistant, αSyn-rich inclusions in neuronal bodies (31).

**Figure 1.**
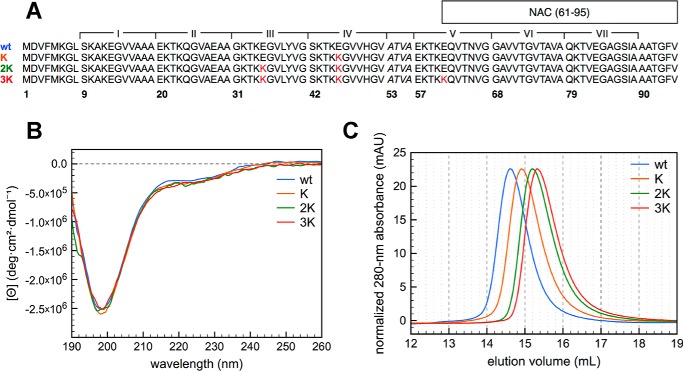
**Structural features of the E46K-like αSyn mutants in the lipid-unbound state.** *A*, primary sequence of the N-terminal region (1–95) of WT, K, 2K, and 3K) αSyn. Glu-to-Lys mutation sites are shown in *red*. The non-amyloid-β component (*NAC*) domain (61–95) and the seven 11-mer repeats *X*KTKEGV*XXXX* are annotated on the sequence. *B*, far-UV CD spectra of 10 μm WT, K, 2K, and 3K αSyn in 10 mm NH_4_Ac (pH 7.4), measured at 25 °C. The single minimum around 200 nm is characteristic of a predominantly unfolded (random coil) secondary structure. *C*, size-exclusion chromatography (Superdex 200 10/300 GL) of the E46K-like αSyn mutants, monitored by 280-nm absorbance (the absorbance signal shown in the chromatogram was normalized to equalize the peak heights). As a reference, an ovalbumin standard (44 kDa) elutes on the same column at 15.375 ml.

In this work we characterize the lipid-binding and lipid-remodeling abilities of three of these Glu-to-Lys mutants (E46K, “K”; E35K,E46K, “2K”; and E35K,E46K,E61K, “3K”; Fig. 1) using a variety of biophysical methods. Through comparisons between multiple E46K-like substitutions in αSyn's N-terminal region, we draw insights into the molecular pathology of E46K (and E46K-like) αSyn mutants and the structure of membrane-bound αSyn.

## Results

### E46K-like mutants increase N-to-C interactions and coil compactness

To characterize whether the Glu-to-Lys mutations induced any change in the structure of the lipid-unbound protein, CD spectra of the recombinant mutants in buffer were measured. Recombinant WT αSyn has long been known to exist in a predominantly unstructured “random coil” conformation (8), and the fPD mutant E46K and the 2K and 3K artificial mutants do not appear to affect the protein secondary structure enough to cause a change in the far-UV CD spectrum (Fig. 1*B*). Analogously, the weak near-UV CD signal of the protein is minimally affected by the mutations, if at all (Fig. S1). Nevertheless, size-exclusion chromatography analysis of the mutants' hydrodynamic radii shows a marked decrease moving from the WT to E46K (as reported previously (32)) and from E46K to 2K and 3K (Fig. 1*C*). Although the elution volumes still indicate that all mutants have an extended conformation (because their hydrodynamic radii are unusually high for 14.5-kDa proteins (8)), the addition of lysines induces increasingly more compact structures.

**Figure 2.**
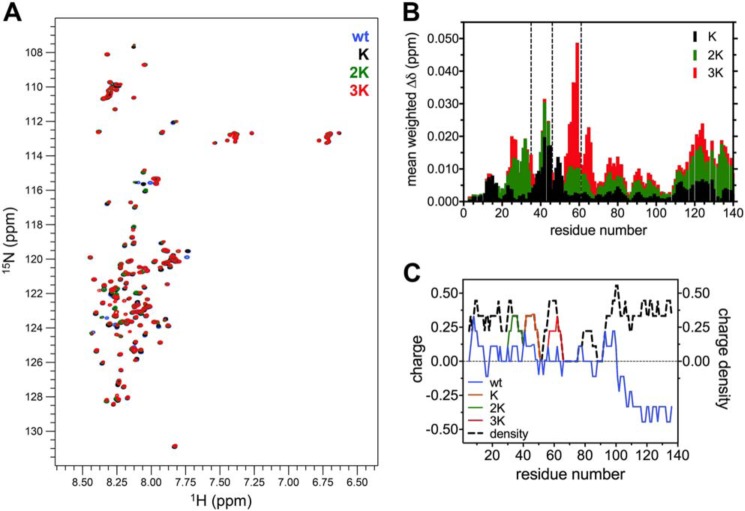
**NMR spectroscopy of the E46K-like αSyn mutants in the lipid-unbound state.** *A*, overlaid ^1^H-^15^N HSQC spectra of 120 μm WT, K, 2K, and 3K αSyn in 50 mm phosphate buffer and 10% D_2_O (pH 6.8), measured at 15 °C and realigned using Ala-140. *B*, histogram of the mean weighted chemical shift perturbations (calculated as [(Δδ^1^H)^2^+(0.15·Δδ^15^N)^2^]^1/2^) of each mutant *versus* WT αSyn, plotted against the corresponding residue number. In addition to the N-terminal region, the C terminus is also affected and shows a clear correlation between the entity of the perturbations and the number of Glu-to-Lys mutations. *C*, charge (and charge density, *i.e.* absolute charge value) distribution of WT αSyn and its mutants, averaged over a window of nine amino acids. The transition from the N-terminal amphipathic region to the negatively charged C terminus can be observed around residue 100. Glu-to-Lys mutations, although not changing the charge density distribution, increase the charge polarization, making electrostatic N-to-C interactions more favorable.

Because CD cannot document small changes in the proteins' conformational equilibria upon Glu-to-Lys substitution, ^1^H-^15^N HSQC spectra of the ^15^N-labeled proteins in buffer (at 15 °C) were also recorded and compared (Fig. 2*A* and Table S1). In accord with the CD data, the overall spread of the amide peaks of the E46K-like mutants reflects their disordered structure. However, numerous shifts in the resonances can be clearly observed, and some of these changes (especially in the case of C-terminal residues) correlate well with the increment in Glu-to-Lys mutations. After assignment of the amide peaks, relying on deposited datasets of the ^1^H-^15^N HSQC of WT αSyn (33, 34), a histogram of the absolute weighted chemical shift perturbations throughout the protein sequence was obtained, highlighting the regions that are mostly affected by the sequential Glu-to-Lys substitutions (Fig. 2*B*). Compared with WT αSyn, all mutations show changes in the N terminus, not only around the site of the Glu-to-Lys substitutions but also mild-, medium-, and long-range effects. Interestingly, the C-terminal region is also markedly affected by the mutations and in a much clearer incremental fashion (*i.e.* correlating with the insertion of more and more lysines) than the N terminus. WT αSyn is known to exist in a variety of unstructured conformational states, many of which are stabilized by long-range N-to-C interactions, probably electrostatic in nature (35, 36). E46K αSyn has also been shown, in both experiments and simulations, to have increased long-range contacts, most likely because of the substitution of the Glu-46 negative charge with a positively charged lysine, making transient bonding with the negative C-terminal region more favorable (37, 38). We can imagine similar (and much more marked, at least according to the magnitude of the perturbations) effects playing a role in this case, explaining the decrease in hydrodynamic radius as well (Fig. 1*C*). Indeed, analysis of the charge distribution of the N terminus of WT αSyn and comparison with its K, 2K, and 3K mutants confirms that, although the overall charge density is unperturbed by the mutations, the Glu-to-Lys substitutions increase the overall charge polarization (Fig. 2*C*). If we take the segment 1–95 as the N terminus of the protein, we observe a net +6 charge increase going from WT αSyn (which has a mild imbalance of +3.1 charges) to 3K αSyn.

### E35K and E46K most affect the micelle-bound “broken helix”

**Figure 3.**
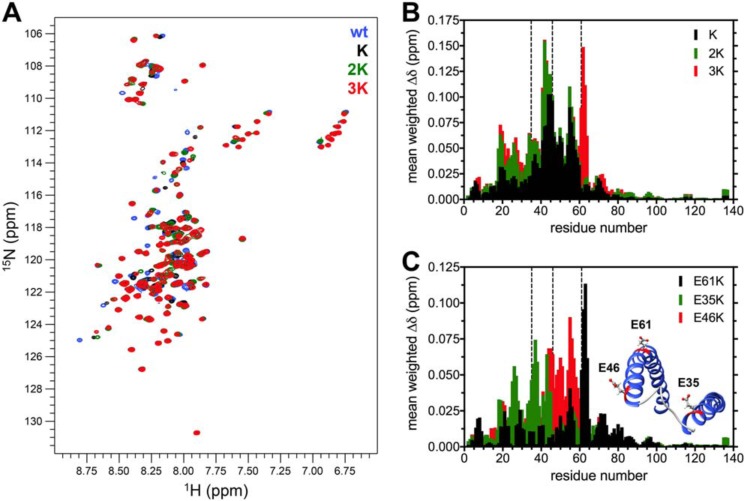
**NMR spectroscopy of the E46K-like αSyn mutants in the SDS-bound state.** *A*, overlaid ^1^H-^15^N HSQC spectra of 120 μm WT, K, 2K, and 3K αSyn in 15 mm SDS, 50 mm phosphate buffer, and 10% D_2_O (pH 6.8), measured at 37 °C and realigned using Ala-140. *B*, histogram of the mean weighted chemical shift perturbations (calculated as [(Δδ^1^H)^2^+(0.15·Δδ^15^N)^2^]^1/2^) of each mutant *versus* WT αSyn, plotted against the corresponding residue number. *C*, histogram of the mean weighted chemical shift perturbations of E46K *versus* WT (*E46K*), 2K *versus* E46K (*E35K*), and 3K *versus* 2K (*E61K*), plotted against the corresponding residue number. Although the E35K and E46K mutations cause wide-ranging perturbations in the N-terminal region of SDS-bound αSyn, the effects of E61K are mostly clustered around the site of the mutation. This is easily understood upon examination of the deposited structure of SDS-bound αSyn (*inset*, PDB code 1XQ8, showing the position and orientation of the glutamates in the sites of Glu-to-Lys mutation).

Extensive biophysical work has concluded that a single continuous helix is the preferred conformation of αSyn upon interaction with curved lipid bilayers (*e.g.* small unilamellar vesicles (SUVs) and large unilamellar vesicles (LUVs)) similar in morphology and composition to the physiological membranous interactants of αSyn (17, 39). Nevertheless, the best-characterized helical structure of αSyn is that classically described as a broken helix (33) and observed mainly in lipoprotein nanoparticles (12) and on detergent micelles (33, 40). We therefore decided to explore how the Glu-to-Lys mutations affected αSyn's broken-helix structure by recording ^1^H-^15^N HSQC spectra of the recombinant ^15^N-labeled mutants in the presence of SDS micelles (Fig. 3*A* and Table S1). The distribution of the amide resonances of all mutants is consistent with a folded structure, and the Glu-to-Lys mutations seem to cause relatively few perturbations in the chemical shifts, which are mainly clustered in the N terminus of the protein, as is clearest after plotting the chemical shift perturbations of each mutant compared with WT αSyn (Fig. 3*B*). By comparing each of the K mutants with its homolog (with one less Glu-to-Lys substitution) we can also probe how each site affects the structure of the broken helix (although in an imperfect fashion, as the perturbations could well not be perfectly additive). These differences are shown in Fig. 3*C*. Although both E46K and E35K have robust, long-ranging effects on αSyn's resonances, E61K appears to act prevalently on a local scale, with only minor “ripples” in the residues away from the substitution site. Inspection of the deposited structure of micelle-bound αSyn (PDB code 1XQ8) allows us to draw structural correlations that confirm our NMR findings. Although Glu-35 and Glu-46 are localized close to the “ordered loop” region and oriented in a way that facilitates electrostatic interactions with the negatively charged heads of the detergent, Glu-61 points directly opposite of the hydrophobic side of the second amphipathic helix and can therefore be expected to only marginally affect the structure of αSyn upon charge inversion (Fig. 3*C*, *inset*). These results contradict previous *in vitro* and *in vivo* work with the E46K-like mutants, which showed a stepwise increase in toxicity with further Glu-to-Lys mutations and, if anything, a more prominent effect of the E61K mutation (28, 31). A structure other than the broken helix could be predominant in cellular and murine models and would explain such inconsistencies.

### Glu-to-Lys mutations mildly increase the “avidity” for curved membranes

**Figure 4.**
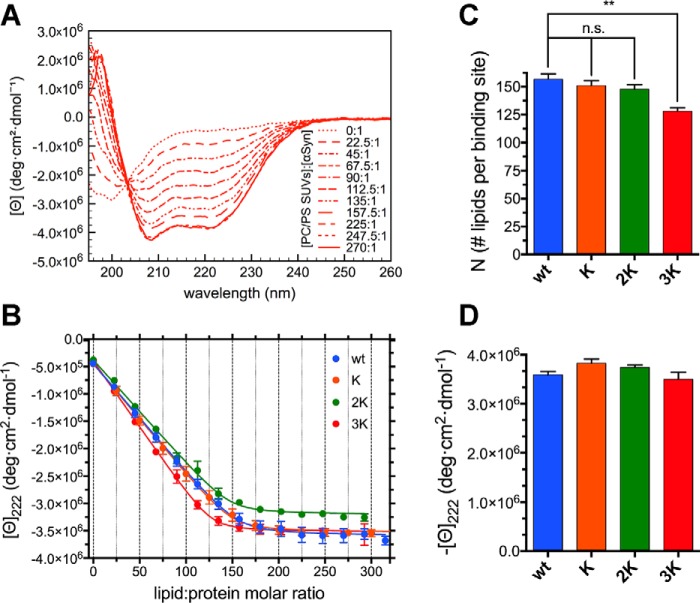
**Glu-to-Lys mutations result in mildly increased αSyn binding to curved charged membranes.** *A*, representative far-UV CD spectra from a titration of 10 μm WT αSyn (in 10 mm NH_4_Ac (pH 7.4)) with 6 mm 70:30 POPC:DOPS SUVs, measured at 25 °C. There is a clear transition from the (almost completely) unstructured lipid-unbound protein to the robustly helical endpoints of the titration (*e.g.* lipid:protein molar ratio 270:1), reflecting the binding and folding of αSyn. *B*, titration curves obtained from raw CD data after extracting the molar ellipticity at 222 nm and graphing it against the lipid:protein molar ratio at each point of the titration. Datapoints are shown along with their standard errors (from *n* = 3 independent titrations) and their best fit with an *N* independent binding sites model (see “Experimental procedures”). *C*, histogram of the *N* values (along with their standard errors, *n* = 3) obtained from the best fit of the titration curves with an *N*-independent binding sites model. Decreasing *N*s indicate increased binding (or avidity, see “Results”). *D*, histogram of the 222-nm plateau molar ellipticity values (along with their standard errors, *n* = 3) obtained from the end-point CD spectra of each mutant after renormalization using the WT αSyn isodichroic point. *n.s.*, *p* > 0.05; **, *p* ≤ 0.01.

To further explore the molecular events leading to the vesicle clustering and toxicity caused by overexpression of the E46K-like mutants (26), we studied the binding of WT αSyn and its Glu-to-Lys mutants to negatively charged SUVs. Upon interaction with curved membranes, such as when titrating the recombinant protein with SUVs, αSyn undergoes a coil-to-helix transition that can be monitored by CD (13). SUVs were prepared by sonication (obtaining a dispersion of vesicles ∼33 nm in diameter; Table S2) of a 70:30 hydrated mixture of zwitterionic 1-palmitoyl-2-oleoylphosphatidylcholine (POPC) and anionic 1,2-dioleoyl-*sn*-glycero-3-phospho-l-serine (DOPS). αSyn, as expected, bound avidly (Fig. 4, *A* and *B*) to these membranes; the titration curves obtained were analyzed to derive the thermodynamic and stoichiometric parameters of the binding event. An *N*-independent binding site model was used to describe the binding events (see “Experimental procedures”). Because the molar lipid concentration was used as the ligand concentration, the *N* value, rather than the number of binding sites, measures the average number of lipid molecules that are necessary to form a lipid binding site for αSyn. As the number of lipid molecules constituting a vesicle can be considered constant, *N* is then inversely proportional to the number of binding sites on a given vesicle. A decrease in *N* therefore reflects a higher number of saturable binding sites on the vesicles' surface and vice versa. We observe a mild decreasing trend in the *N* values obtained by fitting the mutants' titration curves; this represents an increase in the number of αSyn binding sites, although this result does not reach significance in the cases of K and 2K αSyn (Fig. 4*C* and Table S3). Although E46K has been reported previously to have a higher affinity for negatively charged, curved lipid interfaces (41, 42), more recent work has not been able to detect differences with WT αSyn, either when using recombinant E46K αSyn and artificial liposomes or measuring synaptic localization in cultured primary neurons (43). It must also be noted that E61K has the highest effect on *N* of all Glu-to-Lys mutations. Finally, the increase in binding does not correspond to significant changes in the plateau ellipticity values associated with the membrane-bound helical structures, suggesting only minor (if any) conformational rearrangements. Similar CD-monitored lipid-into-protein titrations were also performed in the presence of 150 mm NaCl to test the effect of a higher ionic strength on αSyn's binding behavior and confirmed the results obtained under low-salt conditions (Fig. S2 and Table S3). As expected, the end-point ellipticities and the *k_B_* values are smaller, probably because of the electrostatic masking of the charged moieties in the lipid heads and in some of αSyn's residues, decreasing the strength of ionic interactions at the vesicular interface.

**Figure 5.**
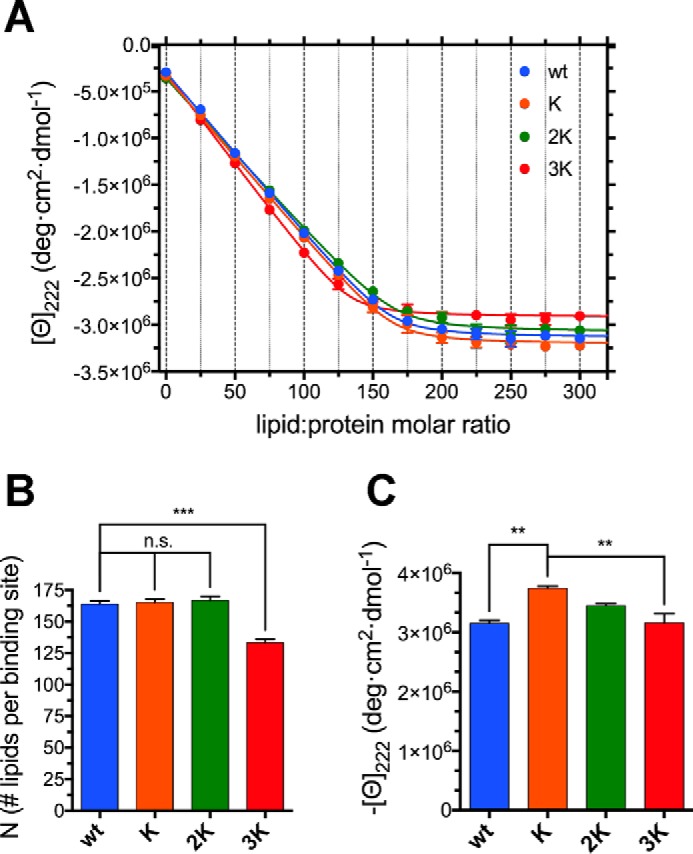
**E61K alone induces mildly increased binding to curved cholesterol-rich membranes.** *A*, titration curves of 10 μm WT, K, 2K, and 3K αSyn (in 10 mm NH_4_Ac (pH 7.4)) with 6 mm 52.5:17.5:30 POPC:DOPS:cholesterol SUVs (measured at 25 °C). Plots were obtained from raw CD data after extracting the molar ellipticity at 222 nm and graphing it against the lipid:protein molar ratio at each point of the titration. Datapoints are shown along with their standard errors (from *n* = 3 independent titrations) and their best fit with an *N*-independent binding site model (see “Experimental procedures”). *B*, histogram of the *N* values (along with their standard errors, *n* = 3) obtained from the best fit of the titration curves with an *N*-independent binding site model. Decreasing *N*s indicate increased binding (or avidity, see “Results”). *C*, histogram of the 222-nm plateau molar ellipticity values (along with their standard errors, *n* = 3) obtained from the end-point CD spectra of each mutant after renormalization using the WT αSyn isodichroic point. *n.s.*, *p* > 0.05; **, *p* ≤ 0.01; ***, *p* ≤ 0.001.

Knowing the critical role of cholesterol-rich lipid rafts in αSyn's physiology (18) and the high content of cholesterol in the membranes of synaptic vesicles (15), we also studied whether the rigidity of the double layer affected the binding of the Glu-to-Lys mutants. CD-monitored titrations were performed in the same way as those with 70:30 POPC:DOPS, but a 52.5:17.5:30 POPC:DOPS:cholesterol lipid dispersion was sonicated to generate SUVs (∼36 nm in diameter). The results (both *N* and the plateau ellipticity values) are similar to those obtained with POPC:DOPS SUVs (Fig. 5), but, in this case, there is a much clearer distinction between the binding behavior of the 3K and that of the other mutants, which are practically indistinguishable and show no trends in their *N* values (Fig. 5*B*). Again, E61K appears to be the mutation site that most significantly affects the ability of αSyn to associate with membranes.

Although CD-monitored lipid-into-protein titrations can provide accurate estimates of the number of lipids per binding site (*N*) and macroscopic structural information regarding the coil-to-helix transition, their estimates of the apparent microscopic binding constants (*k_B_*) are poor. For this reason, and to confirm the previous results by means of an independent technique, we also measured the binding curves of WT αSyn and its Glu-to-Lys mutants to SUVs by isothermal titration calorimetry (ITC). The results for 70:30 POPC:DOPS are analogous to those found by CD-monitored titrations (Fig. S3, *A* and *C*, and Table S3). In addition to *N*, ITC provides thermodynamic information about the heat released upon binding (Fig. S3*D*), but the interpretation of these data, although marked differences are observed between the mutants, is made challenging by the fact that the Δ*H* values are a combination, among others, of the heat of binding of αSyn to the membranes, the heat exchanged because of the coil-to-helix transition, and the heat contributions of the lipids themselves (*e.g.* tail hydration, bilayer rearrangement, and remodeling). ITC, as expected, provides much more accurate estimates of the apparent binding constants, which, however, do not appear to change significantly between mutants (Fig. S3*E* and Table S3). ITC of 52.5:17.5:30 POPC:DOPS:cholesterol SUVs did not detect any binding processes in the cases of WT, K, and 2K αSyn, possibly because the heat exchanges of bilayer reorganization processes masked the heat from the protein's binding and folding. The only mutant that showed a clear sigmoidal profile by ITC was 3K αSyn, again in accord with the CD evidence that the E61K mutation alone increases binding to more rigid membranes (Fig. S3*B* and Table S3).

In conclusion, it is important to consider how, based on the combined CD and ITC data, the αSyn mutants (3K in particular) do not necessarily appear to have a higher “affinity”, in its classic interpretation (higher *k_B_*), for curved charged membranes. Rather, the more Glu-to-Lys mutations are present, the more αSyn molecules seem to be able to bind to a single vesicle, which, to borrow from the nomenclature of polyvalent interactions, corresponds to a higher avidity of lipid bilayers for the E46K-like mutants (44).

### Glu-to-Lys mutations cause a progressive loss of curvature selectivity

**Figure 6.**
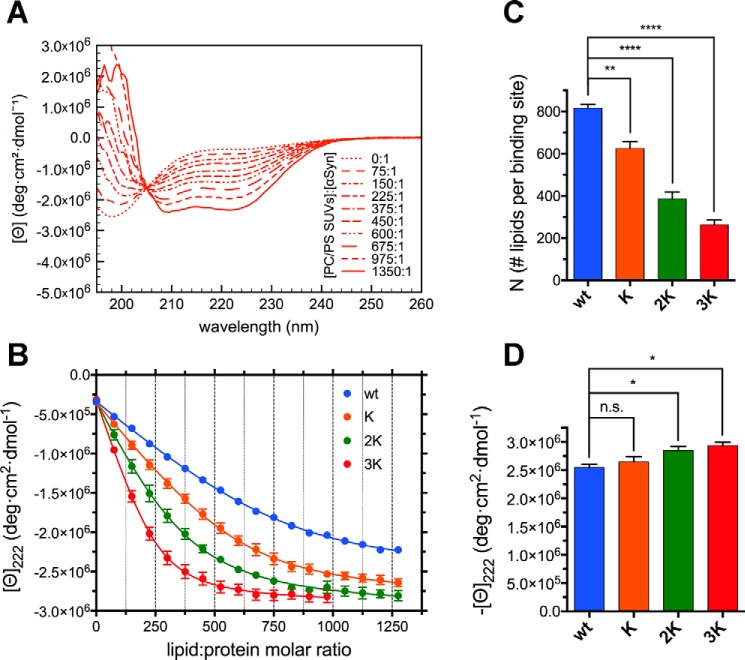
**αSyn's Glu-to-Lys mutations cause a progressive loss of curvature selectivity.** *A*, representative far-UV CD spectra from a titration of 10 μm WT αSyn (in 10 mm NH_4_Ac (pH 7.4)) with 10 mm 70:30 POPC:DOPS LUVs, measured at 25 °C. Although a coil-to-helix transition is also observed in the presence of LUVs, both its sharpness and the entity of the conformational rearrangement are much less than those observed with SUVs (Fig. 4). *B*, titration curves obtained from raw CD data after extracting the molar ellipticity at 222 nm and graphing it against the lipid:protein molar ratio at each point of the titration. Datapoints are shown along with their standard errors (from *n* = 3 independent titrations) and their best fit with an *N*-independent binding site model (see “Experimental procedures”). *C*, histogram of the *N* values (along with their standard errors, *n* = 3) obtained from the best fit of the titration curves with an *N*-independent binding site model. Decreasing *N*s indicate increased binding (or avidity, see “Results”). *D*, histogram of the 222-nm plateau molar ellipticity values (along with their standard errors, *n* = 3) obtained from the best fit of the titration curves after renormalization using the WT αSyn isodichroic point. *n.s.*, *p* > 0.05; *, *p* ≤ 0.05; **, *p* ≤ 0.01; ****, *p* ≤ 0.0001.

One rarely explored aspect of the molecular pathology of fPD-associated αSyn mutants is how their binding selectivity is affected by mutations. Because αSyn has long been known to bind preferentially to small vesicles (13), and its dynamic association with synaptic vesicles upon exo- and endocytosis is believed to be central to its function (19, 45), we chose to explore how Glu-to-Lys mutations modify αSyn's selectivity for certain curvature radii. We performed CD-monitored titrations of WT αSyn and its Glu-to-Lys mutants with LUVs prepared by extrusion of a 70:30 POPC:DOPS dispersion using 0.1-μm polycarbonate membranes, obtaining vesicles ∼100 nm in diameter (Table S2). As expected, the binding propensity of both WT αSyn and its mutants to charged LUVs is markedly lower than that observed with SUVs (Fig. 6, *A* and *B*). The curvature of the titration curves is much shallower than the sharp bends of SUVs titrations, signaling that the *k_B_* values are orders of magnitude lower (Table S3). The ellipticities of the helical conformers of LUV-bound αSyn are also roughly half of those recorded before (Figs. 6*A* and 4*A*), possibly because of a more dynamic equilibrium between partly folded and partly unfolded conformations, which has been proposed to be one of the curvature-sensing mechanisms adopted by αSyn (46). Furthermore, although the effects of Glu-to-Lys mutations are generally mild in the SUV titrations (possibly because of the overall high affinities), robust changes are evident in the LUV titrations, especially after curve fitting with our *N*-independent binding site model (Fig. 6, *C* and *D*). Going from WT αSyn to 3K, we see a decrease in *N* (number of lipids per binding site) that is truly stepwise incremental, as are the effects observed in cellular models (28). In addition, although we could not detect significant differences between the avidity (nor the affinity) of E46K and WT αSyn using SUVs, here we observe a clear loss of curvature selectivity with the introduction of the fPD-causing mutation, which could be one of the factors underlying its propensity for pathology. In addition to the increased avidity of negatively charged LUVs for E46K-like mutants, a slight increase in plateau ellipticities is also detected, but, although significant, the effects are as minor as those described previously in the case of SUVs (Fig. 6*D*).

As with the SUVs titrations, the experiments were repeated in the presence of 150 mm NaCl to explore the effect of increased ionic strength (Fig. S4). However, given the already weak affinity of αSyn for the mildly charged LUVs, the presence of a higher salt concentration almost completely abolished binding for all mutants except 3K (confirming its much stronger ability to bind bilayers in all of the conditions studied). To be able to quantitatively compare the different Glu-to-Lys mutants, we used the slope of the binding curve in its early phase as a measure of the strength of the affinity (Fig. S4*C*). Although not as mechanistically valuable as the *N*-independent binding site model, comparison of the binding slopes among the different mutants still shows a much stronger progression (WT to 3K) than the one observed with SUVs (Fig. S4*D*), confirming previous conclusions.

### E46K-like mutants have increasing membrane remodeling abilities

**Figure 7.**
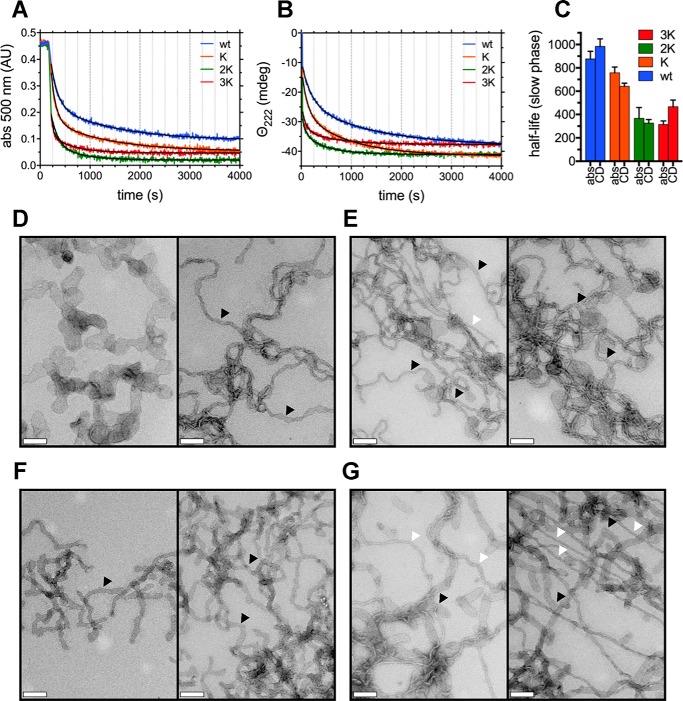
**αSyn's Glu-to-Lys mutations result in increased membrane remodeling.** *A*, vesicle clearance experiments, monitored by absorbance at 500 nm of 400 mm 90:10 POPG:POPC MLV dispersions by 40 μm WT αSyn and its E46K-like mutants (lipid:protein molar ratio 10:1). Best fits were obtained using a two-phase exponential decay model (Table S3) and are shown in *black. B*, vesicle clearance experiments, monitored by CD (222-nm ellipticity), of 400 mm 90:10 POPG:POPC MLV dispersions by 40 μm WT αSyn and its E46K-like mutants (lipid:protein molar ratio 10:1). Best fits were obtained as in *A* and are shown in *black* (Table S3). *C*, histogram of the (slow-phase) half-life values (along with their 95% confidence interval) obtained from the best fit of the clearance kinetics in *A* and *B* with a two-phase exponential decay model. As more Glu-to-Lys mutations are introduced, the clearance rate increases (and, thus, half-lives decrease), but the trend plateaus at 3K αSyn. *D–G*, electron micrographs, negatively stained, of lipid tubule dispersions generated by mixing WT (*D*), E46K (*E*), 2K (*F*), and 3K (*G*) αSyn to 90:10 POPG:POPC MLV preparations. *Scale bars* = 200 nm. Although all mutants present the ability to robustly tubulate membranes, with progressive Glu-to-Lys mutations, the number of large, vesicular-like “cisternae” (*e.g. D*, *left*) and bilayer tubes (*black arrowheads*) formed upon incubation with αSyn decreases, and thinner, micellar (*e.g. G*, *right*) tubes can be observed (*white arrowheads*). *abs*, absorbance.

One further aspect of αSyn's interplay with membranes was then studied; namely, its ability to remodel lipid bilayers into bilayer tubes, micellar tubes, and lipoprotein nanoparticles (12, 47, 48). A dispersion of multilamellar vesicles (MLVs) was prepared by hydration of a 90:10 mixture of 1-palmitoyl-2-oleoyl-*sn*-glycero-3-phospho-(1′-*rac*-glycerol) (16:0–18:1 PG or POPG) and POPC. The content of charged lipids was chosen to magnify differences in tubulation rates arising from progressive Glu-to-Lys mutations. Upon addition of αSyn, the robust scattering signal from the heterogeneous dispersion of large (*d* > 1 μm) MLVs is drastically decreased as αSyn induces the formation of tubules, vesicular structures, and lipoprotein nanoparticles, which all exhibit reduced scatter. Remodeling rates can thus be studied by light scattering (*e.g.* absorbance at 500 nm) and also by CD because it is the association of αSyn with the vesicles (and its consequent folding) that drives the membrane remodeling (Fig. 7, *A* and *B*). The rates of vesicle clearance correlate well with αSyn's increasing avidity for LUVs, with a stepwise increase going from WT to 2K αSyn; however, they plateau at 3K, showing little difference upon introduction of E61K. Fitting of the clearance curves using a two-phase exponential decay confirms qualitative observations (the decay rates of the “slow” phase were compared, because the early “fast” phase has too few points to provide accurate parameters). Half-lives decrease upon introduction of the first two Glu-to-Lys mutations (E46K and E35K), and no differences can be detected between 2K and 3K (both in absorbance-monitored and CD-monitored experiments; Fig. 7*C*). Electron micrographs obtained after mixing WT αSyn and its Glu-to-Lys mutants with MLVs also reveal robust membrane remodeling in all cases. However, the introduction of successive Glu-to-Lys mutations leads to the more frequent appearances of narrower (*d* ∼ 5–6 nm) membrane tubes (Fig. 7, *D–G*). Previous work (47, 48) has shown how such narrow tubes are micellar in nature and are increasingly generated at higher protein-to-lipid ratios. This phenomenon reflects the increased avidity of Glu-to-Lys mutants toward large curved membranes (Fig. 6), resulting in a greater number of bound αSyn molecules and more profound bilayer remodeling.

## Discussion

In the search for the molecular determinants underlying the inception of PD and other synucleinopathies, a great deal of attention has been devoted to the biophysics and biochemistry of fPD-associated αSyn mutants. Historically, most of these mutants were characterized in their ability to generate toxic oligomers upon aggregation (49, 50). However, after the discovery of the robust and specific lipid-binding ability of WT αSyn (13), several lines of evidence have shown how disruption of such binding equilibria can also lead to cellular toxicity and disease (20, 24, 51).

In this work, we focused on the molecular mechanism behind the lipid-mediated toxicity of E46K αSyn and its exacerbated analogs (2K and 3K αSyn, Fig. 1*A*), which have been shown, in both cellular and murine models, to cause trafficking defects, αSyn-rich vesicular inclusions, and a PD-like motor phenotype (26, 28, 31). We described how E46K, 2K, and 3K αSyn, in accord with previous reports (26, 31), exhibit a stepwise increase in membrane avidity (Figs. 4–6) and in their membrane-remodeling abilities (Fig. 7). Although the effects of E46K and E46K-like mutations on αSyn's binding to small curved vesicles were relatively minor (Figs. 4 and 5), these mutants caused a much more profound disruption in the protein's curvature selectivity, which was almost completely abolished in 3K αSyn (Fig. 6). Although some reports have previously described E46K αSyn as binding more avidly to small, charged vesicles (41, 42), our tightly controlled CD-monitored titration experiments, combined with an independent technique such as ITC, failed to reproduce these results, in agreement with more recent work (43). 2K and 3K αSyn also displayed only small (or even undetectable) effects when SUVs were used as their binding partners (Figs. 4 and 5) but exhibited much more severe changes in their LUV-binding ability (Fig. 6), strengthening the case that they behave as exacerbated E46K mutants. Finally, membrane tubulation assays, performed using heterogeneous dispersions of large MLVs, confirmed such trends (Fig. 7). Successive Glu-to-Lys mutations appear to transform αSyn from being predominantly a “curvature sensor” (WT) to acting as a robust “curvature inducer” (2K and 3K), although it is likely that these aspects are ultimately linked and can both be traced back to the loss of curvature selectivity.

We hypothesize that behind the pathogenesis of both E46K-associated fPD and 2K/3K murine models of synucleinopathy there could be a redistribution of the membrane-bound αSyn population to larger intracellular vesicles (such as those involved in secretory or endosomal pathways) rather than an increased avidity for synaptic vesicles, their natural binding partners. Such a shift could then act as a combined toxic gain of function, with αSyn disrupting vesicular trafficking through its abnormal binding and damaging loss of function because of αSyn being less available at the synaptic bouton, where it would normally regulate synaptic vesicle fusion and recycling (6).

**Figure 8.**
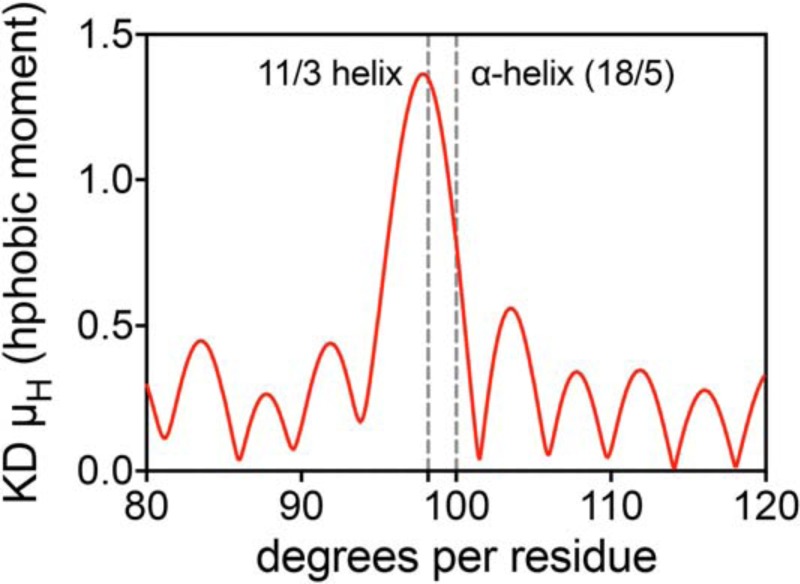
**αSyn's N-terminal region has an intrinsic propensity toward an 11/3 helix.** Shown is the hydrophobic moment, according to Eisenberg's definition (52), of the N-terminal segment of αSyn (1–95), calculated for different helix periodicities (using the hydropathy index values of Kyte and Doolittle (64)) and graphed against the angle between consecutive amino acids. The angles corresponding to an ideal α-helix (100°) and an ideal 11/3 helix (98.18°) are marked and labeled, showing how the amino acid arrangement in the N terminus of αSyn leans toward an 11/3 arrangement if amphiphilicity (*i.e.* segregation of polar and apolar residues) is to be maximized.

In addition to characterizing the molecular pathology of E46K-like mutants, the sequential insertion of Glu-to-Lys mutations at specific points in the 11-mer repeat region allows us to rationalize the observed phenomena using structural determinants. αSyn's primary structure, upon inspection of its sequence hydrophobicity (Fig. S5*A*), is clearly split between a neutral (neither robustly polar nor apolar) N terminus (1–95) and a negatively charged C terminus, consistently characterized as disordered. Also, either by plotting Eisenberg's hydrophobic moment (measuring the degree of polarity segregation in a helical structure) *versus* the helix's periodicity (52, 53) or by calculating the fast Fourier transform of the vector of the side-chain hydrophobicities in segment 1–95 (54, 55), we can see how the N terminus can be arranged into robustly amphiphilic helical structures with periodicities around 3.6 amino acids per turn (Fig. 8 and S5*B*). Depending on the amphiphilic interactant, αSyn's membrane-bound (or detergent-bound) structure has been either described as a broken helix, with two α-helical segments (3.6 amino acids per turn) connected by an ordered loop (33, 40), or as a single continuous 11/3 helix (∼3.67 amino acids per turn), spanning throughout the whole 1–95 region (17, 39). Subsequent work has described the possibility of an interchange between these two forms, quantifying the equilibrium as shifted toward the 11/3 helix when αSyn is bound to charged SUVs rather than detergent micelles (12, 56).

If we plot a Schiffer–Edmundson helical wheel diagram (57) with an 11/3 periodicity and its helical net (58), spanning αSyn's N-terminal region (1–95), and highlight the positions of the three Glu residues progressively substituted by lysines, we can hypothesize how their arrangement could increase their avidity for bilayers with a progression comparable with that observed in our experiments (Fig. S6). The proximity of Glu-35, Glu-46, and Glu-61 to the negatively charged surface of the vesicle's membrane could allow the formation of ionic bridges between the positively charged lysines and the phosphate heads, favoring the binding and stabilizing the 11/3 helix. Although all three residues are in very similar positions, the presence of the 53–56 (ATVA, Fig. 1*A*) break in the 11-mer periodicity region shifts Glu-61 slightly closer to the membrane surface. While this small difference might be masked in 3K αSyn by the dynamic nature of the 11/3 helix (17, 46, 59), especially in fluid POPC/DOPS SUVs (Fig. 4), it could become more important in the case of rigid membranes, rich in cholesterol, also explaining the lone increased avidity of 3K for such interfaces (Fig. 5).

In conclusion, our biophysical characterization recapitulates all relevant features of cellular and murine models of the E46K-like mutants and, at the same time, allows us to advance the possibility of a loss of curvature selectivity, rather than increased membrane affinity, as the critical dyshomeostasis in synucleinopathies. In light of these results, there appears to be an even greater translational potential in therapies modulating the cellular lipid composition to compensate for αSyn defects, which has recently started to be explored (60, 61).

## Experimental procedures

### Materials

1-palmitoyl-2-oleoyl-glycero-3-phosphocholine (16:0–18:1 PC or POPC), 1,2-dioleoyl-*sn*-glycero-3-phospho-l-serine (18:1 PS or DOPS), and 1-palmitoyl-2-oleoyl-*sn*-glycero-3-phospho-(1′-*rac*-glycerol) (16:0–18:1 PG or POPG) were purchased from Avanti Polar Lipids (Alabaster, AL) as chloroform solutions. All other chemicals were obtained from Sigma-Aldrich (St. Louis, MO) unless otherwise noted.

### Molecular cloning

pET21a-α-synuclein, encoding codon-optimized untagged human αSyn, was a gift from the Michael J. Fox Foundation (Addgene plasmid 51486). Fragments encoding E46K (K), E35K,E46K (2K), and E35K,E46K,E61K (3K) αSyn were amplified with PfuUltra II Fusion HS (Agilent Technologies, Santa Clara, CA) using their constructs in pcDNA4 (28) as templates and the primers αSyn_InFu_FWD 5′-AAGGAGATATACATATGGATGTATTCATGAAAGGACTTTCAAAGG-3′ and αSyn_InFu_REV 5′-TGCTCGAGTGCGGCCGCTCAGGCTTCAGGTTCGTAGTCTTGATACC-3′. The amplified fragments were then inserted into pET21a (linearized with NdeI/NotI) using the In-Fusion HD Cloning Kit (Takara Bio, Mountain View, CA), following the manufacturer's protocol. Cloning of pET21a-K-αSyn, pET21a-2K-αSyn, and pET21a-3K-αSyn was confirmed by DNA sequencing and restriction analysis.

### Protein expression and purification

BL21(DE3) *Escherichia coli* (New England Biolabs, Ipswich, MA) were transformed with the pET21a-based constructs, and single colonies were inoculated in LB + ampicillin. Cultures were induced at an OD_600_ of ∼0.5 with 1 mm isopropyl 1-thio-β-d-galactopyranoside (IPTG) for 4 h. The cell pellet, after harvesting, was resuspended in 20 mm Tris buffer and 25 mm NaCl (pH 8) and lysed by boiling for 15 min. The supernatant of a 20-min., 20,000 × *g* spin of the lysate was then processed further. The sample was loaded on two 5-ml (tandem) HiTrap Q HP anion exchange columns (GE Healthcare) equilibrated with 20 mm Tris buffer and 25 mm NaCl (pH 8). αSyn was eluted from the columns with a 25–1000 mm NaCl gradient of 20 mm Tris buffer and 1 m NaCl (pH 8). Peak fractions were pooled and further purified via gel filtration on a HiPrep Sephacryl S-200 HR 16/60 gel filtration column (GE Healthcare) using 50 mm NH_4_Ac (pH 7.4) as running buffer. Peak fractions (>95% pure, by Coomassie Brilliant Blue–stained SDS-PAGE) were pooled, aliquoted, lyophilized, and stored at −20 °C. Purified samples were also analyzed by MALDI-TOF MS and by trypsin digestion followed by MALDI-TOF MS to confirm the intact mass and sequence of the proteins (Molecular Biology Core Facilities, Dana-Farber Cancer Institute). Lyophilized proteins were reconstituted fresh, before experiments, in 10 mm NH_4_Ac (pH 7.4) and not reused (relyophilized and reconstituted) more than twice. Protein solutions were spun down every time after resuspension, before spectroscopic quantitation of the protein concentration, at 21,130 × *g* for 20 min at 4 °C to remove any large αSyn aggregates. Concentration was quantified by measuring the absorbance at 280 nm (ϵ = 0.412 mg·ml^−1^·cm^−1^).

### ^15^N-labeled protein expression

For the expression of ^15^N-labeled αSyn, the bacteria were grown in M9 complete medium + ampicillin supplemented with [^15^N]H_4_Cl. M9 complete medium was prepared fresh from a 10× M9 medium stock (60 g·L^−1^ Na_2_HPO_4_, 30 g·L^−1^ KH_2_PO_4_, 5 g·L^−1^ NaCl), 50× 20% d-glucose, 1000× 1 m MgSO_4_, 5000× 1 m CaCl_2_, 1000× 1 mg·ml^−1^ thiamine and biotin, 100× trace element solution, 1000× vitamin solution, and 1 g·L^−1^ [^15^N]H_4_Cl. The 100× trace element solution was prepared by dissolving (in 1 liter of MilliQ water) 5 g of EDTA, 0.50 g of FeCl_3_, 84 mg of ZnCl_2_, 12 mg of CuSO_4_, 10 mg of CoCl_2_·6H_2_O, 10 mg of H_3_BO_3_, 1.6 mg of MnCl_2_·4H_2_O, and 1.6 mg of Na_2_MoO_4_·2H_2_O and adjusting the pH to 7.0 (it was then filter-sterilized and stored at room temperature). The 1000× vitamin solution was prepared by dissolving (in 1 liter of 95% MilliQ water and 5% acetonitrile) 50 mg of riboflavin, 0.5 g of niacinamide, 0.5 g of pyridoxine hydrochloride, and 0.5 g of thiamine. After filtration with a 0.22-μm cutoff (to sterilize and remove the undissolved riboflavin), the solution was stored at 4 °C protected from light. BL21(DE3) *E. coli* (New England Biolabs) were transformed with the pET21a-based constructs and single colonies inoculated in 2× YT + ampicillin. Cultures were grown for 6–8 h. at 37 °C under shaking and then used to inoculate an overnight preculture in M9 complete medium + ampicillin, which, on the following day, was diluted again 1:10 in M9 complete medium + ampicillin. The expression and purification of ^15^N-labeled proteins were, from this point on, performed as described under “Protein expression and purification.”

### Size-exclusion chromatography

Samples were injected on a Superdex 200 (10/300 GL) column (GE Healthcare) at room temperature and eluted with 50 mm NH_4_Ac (pH 7.4) while measuring (in-line) the conductivity and the 280-nm absorption of the eluate. For size estimation, a gel filtration standard (catalog no. 151-1901, Bio-Rad) was run on the column, and the calibration curve was obtained by semi-logarithmic plotting of molecular weight *versus* the elution volume divided by the void volume.

### Liposome preparation

Aliquots from chloroform stock solutions of POPC, DOPS, and cholesterol were mixed thoroughly in the desired proportions and dried under a stream of argon. The dried lipid film was then kept under a vacuum overnight to remove traces of organic solvent. For both SUVs and LUVs, lipid solutions were freshly prepared by resuspending the dried lipids in 10 mm NH_4_Ac (pH 7.4) to their final concentrations and hydrating them for 1 h at 37 °C while nutating. SUVs were prepared by pulse-sonicating the phospholipid suspensions for 10–20 min at room temperature with a microtip sonicator (Misonix S-4000, Qsonica, Newtown, CT). The SUV suspensions were then spun down at 21,130 *xg* for 10 min to remove any metal particles and larger lipid aggregates. LUVs were prepared by manual extrusion (25 passes) using an Avanti Mini-Extruder (Avanti Polar Lipids) and 0.1-μm polycarbonate membranes, following the manufacturer's protocol. For MLVs, aliquots from the lipid stock solutions in chloroform were mixed thoroughly in the desired proportions and dried under a stream of nitrogen. The dried lipid film was then kept under vacuum for more than 12 h to remove traces of organic solvent. MLV dispersions were prepared by resuspending the dried lipids in the buffer of choice and vortexing.

### CD

Spectra were recorded with a Jasco J-815 spectropolarimeter (Jasco, Easton, MD) at 25 °C using a 1-mm path length cuvette for far-UV CD (a 1-cm path length cuvette was used for near-UV CD measurements). Temperature control with an accuracy of ±0.1 °C was achieved with a heating/cooling accessory equipped with a Peltier element (PFD-425S) connected to a water thermostatic bath. When possible, buffer spectra were recorded and subtracted. In titration experiments, spectra were corrected for the dilution effect from the addition of titrant; control lipid-into-buffer titrations were also recorded to confirm the negligibility of the liposome scattering signal. Three independent titrations (although with the same vesicle preparation) were performed for each condition. In “high-salt” (150 mm NaCl) titrations, NaCl was spiked into protein and lipid samples right before the experiments using a 5 m stock.

Stoichiometric and thermodynamic parameters (*N*, ϴ, *k_B_*) were determined by fitting the CD titration curves according to an *N*-independent binding site model. The expression of the fractional saturation ϑ (ratio of occupied sites to total sites) as a function of *P_t_* and *L_t_* (the total concentrations of protein and lipid), *k_B_* (the apparent microscopic binding constant), and *N* (the number of saturable binding sites on the surface of the vesicles) was taken from the classic derivation of the Wiseman isotherm for an *N*-independent binding site model (62).

(Eq. 1) $$\theta =\frac{1}{2}\left[1+\frac{L_{t}}{NP_{t}}+\frac{1}{Nk_{B}P_{t}}\right]-\sqrt{{\left(1+\frac{L_{t}}{NP_{t}}+\frac{1}{Nk_{B}P_{t}}\right)}^{2}-\frac{4L_{t}}{NP_{t}}}$$

In CD-monitored titrations, the 222-nm signal comes from the combination of the random coil signal from unbound αSyn and the helical signal of lipid-bound αSyn, each proportional to the molar ratios of the two species (ϴ is the molar, or residue, ellipticity at each point of the titration).

(Eq. 2) $${\left[\theta \right]}_{i}^{222}=\frac{\left[PL_{n}\right]}{P_{t}}{\left[\theta \right]}_{helix}^{222}+\frac{\left[P\right]}{P_{t}}{\left[\theta \right]}_{coil}^{222}$$

ϑ (at each point of the titration) can then be expressed as

(Eq. 3) $$\frac{\left[PL_{n}\right]}{P_{t}}=\theta =\frac{{\left[\theta \right]}_{i}^{222}-{\left[\theta \right]}_{coil}^{222}}{{\left[\theta \right]}_{helix}^{222}-{\left[\theta \right]}_{coil}^{222}}$$

Combining Equations 1 and 3, we can write ϴ as a function of the lipid:protein molar ratio *X*, obtaining the final form of the fitting equation.

(Eq. 4) $${\left[\theta \right]}_{i}^{222}={\left[\theta \right]}_{coil}^{222}+\frac{{\left[\theta \right]}_{helix}^{222}-{\left[\theta \right]}_{coil}^{222}}{2}\left[1+\frac{X}{N}+\frac{1}{Nk_{B}P_{t}}-\sqrt{{\left(1+\frac{X}{N}+\frac{1}{Nk_{B}P_{t}}\right)}^{2}-\frac{4X}{N}}\right]$$

Because the molar lipid concentration in the cuvette is used as *L_t_*, the *N* value, rather than the number of binding sites, measures the average number of lipid molecules forming one lipid binding site.

### ITC

ITC measurements were performed on an iTC200 instrument (Malvern Instruments, Westborough, MA). Typically, 18 fresh SUV dispersion injections of 2 μl each (+1 0.4-μl preinjection) were titrated in the calorimeter chamber. Both linear baselines and control titrations of vesicles into buffer alone were used for baseline subtraction. The data were processed using the MicroCal ORIGIN 7.0 software (Malvern Instruments, OriginLab, Northampton, MA). After integration of the differential power signal, stoichiometric and thermodynamic parameters (*N*, Δ*H*, and *k_B_*) were determined by fitting the sigmoidal titration curve using an *N*-independent binding site model (with the sites assumed to localize on the vesicles' outer surface). The apparent (given the approximations used) microscopic binding constant was labeled *k_B_*. The total lipid concentration in the syringe was taken as the ligand concentration; thus, the *N* value measures the average number of lipid molecules forming a binding site. In all experiments, the stoichiometric and thermodynamic parameters were obtained from three independent titrations (although performed using the same vesicle preparation). Δ*S* was obtained from Δ*G* = −*RTlnk_B_* and Δ*H.*

### Dynamic light scattering

The hydrodynamic radius of the liposomes (5 mm) was measured using a DynaPro dynamic light scattering instrument (Wyatt Technology Corp., Santa Barbara, CA). The dynamic light-scattering cell was thermostated at 20.0 °C; the hydrodynamic radii were obtained by averaging three independent samples.

### Phospholipid vesicle clearance assay

The ability of αSyn and its mutants to remodel phospholipid vesicles was studied by measuring light scattering as a function of time using a Jasco V-550 UV/VIS spectrophotometer as described previously (47). Briefly, light scattering was monitored at 500 nm using a 2-nm slit width and a “medium” response time. MLVs were prepared in 20 mm HEPES and 100 mm NaCl (pH 7.4). No changes in light scattering of the MLV dispersion were observed in the absence of protein. CD-monitored vesicle clearance experiments were performed at 25 °C using a Jasco J-810 spectropolarimeter in a 1-mm quartz cell. Blanks were recorded under similar conditions and subtracted.

### Transmission electron microscopy

Transmission electron microscopy specimens were prepared on carbon-coated formvar grids (Electron Microscopy Sciences, Hatfield, PA). Samples were adsorbed on the grids for 5 min and then negatively stained with 1% (w/v) aqueous uranyl acetate. Images were taken on a JEOL 1400 transmission electron microscope (JEOL USA Inc., Peabody, MA) at an accelerating voltage of 100 kV.

### NMR

^1^H-^15^N HSQC spectra of ^15^N-labeled αSyn were recorded in 50 mm phosphate buffer (pH 6.8) and 10% D_2_O. Spectra were recorded on a 600 MHz Bruker AVANCE II spectrometer (Bruker, Billerica, MA) equipped with a Prodigy CryoProbe at either 15 °C (120 μm αSyn) or 37 °C (120 μm αSyn and 15 mm SDS). Resonances were assigned, where possible, by inspection and comparison with the previously assigned spectra of WT αSyn (BMRB 5744, 6968) (33, 34). Data processing and analysis were performed with Bruker TopSpin software and CcpNmr Analysis (63). Perturbations in the chemical shift values for ^1^H and ^15^N were calculated as [(Δδ^1^H)^2^ + (0.15·Δδ^15^N)^2^]^1/2^.

### Data analysis and primary sequence analysis

Data analysis was performed using GraphPad Prism 7 (GraphPad Software, La Jolla, CA). Statistical significance was determined by ordinary one-way analysis of variance followed by Sidak's multiple comparisons test, with a single pooled variance: n.s., *p* > 0.05; *, *p* ≤ 0.05; **, *p* ≤ 0.01; ***, *p* ≤ 0.001; ****, *p* ≤ 0.0001.

Primary sequence analyses were performed with Microsoft Excel (Microsoft, Redmond, WA) and Wolfram Mathematica 10 (Wolfram Research, Champaign, IL), using the hydropathy index values of Kyte and Doolittle (64). Helical wheel and net diagrams were plotted using in-house Python code, modeled after the WHEEL and HELNET programs (65).

## Author contributions

M. R., A. E. P., H. J., J. C. P., L. F.-O., and D. S. P. data curation; M. R., A. E. P., H. J., J. C. P., L. F.-O., A. A., R. L., J. V., and T. B. formal analysis; M. R., A. E. P., H. J., J. C. P., L. F.-O., and D. S. P. investigation; M. R., A. E. P., H. J., J. C. P., L. F.-O., and A. A. visualization; M. R., J. V., and T. B. writing-original draft; M. R., A. E. P., H. J., J. C. P., L. F.-O., D. S. P., A. A., R. L., J. V., and T. B. writing-review and editing; D. S. P. validation; D. S. P. methodology; A. A. software; R. L., J. V., and T. B. conceptualization; R. L., J. V., and T. B. supervision; R. L., J. V., and T. B. funding acquisition.

## Supplementary Material

Supporting Information

## Footnotes

3 The abbreviations used are:

PD: Parkinson's disease
αSyn: α-synuclein
fPD: familial Parkinson's disease
HSQC: heteronuclear single-quantum coherence
SUV: small unilamellar vesicle
LUV: large unilamellar vesicle
POPC: 1-palmitoyl-2-oleoyl-glycero-3-phosphocholine
DOPS: 1,2-dioleoyl-*sn*-glycero-3-phospho-l-serine
ITC: isothermal titration calorimetry
MLV: multilamellar vesicle
POPG: 1-palmitoyl-2-oleoyl-*sn*-glycero-3-phospho-(1′-*rac*-glycerol).
