# Supplementary material for: E46K-like α-synuclein mutants increase lipid interactions and disrupt membrane selectivity
Source: J Biol Chem. 2019 May 2;294(25):9799–812. doi: 10.1074/jbc.RA118.006551 (PMC6597829; doi:10.1074/jbc.RA118.006551)
Supplement: Supporting Information [file supp_294_25_9799__index.html]

E46K-like α-synuclein mutants increase lipid interactions and disrupt membrane selectivity — E46K-like α-synuclein mutants disrupt membrane selectivity — Supporting Information 

# E46K-like α-synuclein mutants increase lipid interactions and disrupt membrane selectivity

## Supporting Information

- Supporting Information - Figures S1, S4, S5, S6, S7, S8 and Tables S2, S3.
- Supporting Information (to be published online) -

  Table S1.
