# Supplementary material for: E46K-like α-synuclein mutants increase lipid interactions and disrupt membrane selectivity
Source: J Biol Chem. 2019 May 2;294(25):9799–812. doi: 10.1074/jbc.RA118.006551 (PMC6597829; doi:10.1074/jbc.RA118.006551)
Supplement: Supporting Information [file supp_RA118.006551_141724_2_supp_323605_pqtlkz.pdf]

SUPPORTING INFORMATION: E46K-like α-synuclein mutants increase lipid interactions and disrupt membrane selectivity

Matteo Rovere[1], Alex E. Powers[1], Julia C. Pitino[1], Luis Fonseca-Ornelas[1], Dushyant S. Patel[1], Alessandro Achille[2], Ralf Langen[3], Jobin Varkey[3], Tim Bartels[1,4]*.

From the [1]Ann Romney Center for Neurologic Diseases, Brigham and Women's Hospital and Harvard Medical School, Boston MA, 02115, USA; the [2]Department of Computer Science, University of California, Los Angeles, Los Angeles CA, 90095, USA; the [3]Zilkha Neurogenetic Institute, University of Southern California, Los Angeles CA, 90033, USA; [4]the Dementia Research Institute, University College London, London, WC1E 6BT, UK

*To whom correspondence should be addressed: Dementia Research Institute, University College London, London, Gower Street, Cruciform Building Wing 2.4, WC1E 6BT, UK. Phone: +44-20-31086814; E-mail: t.bartels@ucl.ac.uk

Figures S1–S6 and Tables S1–S3.

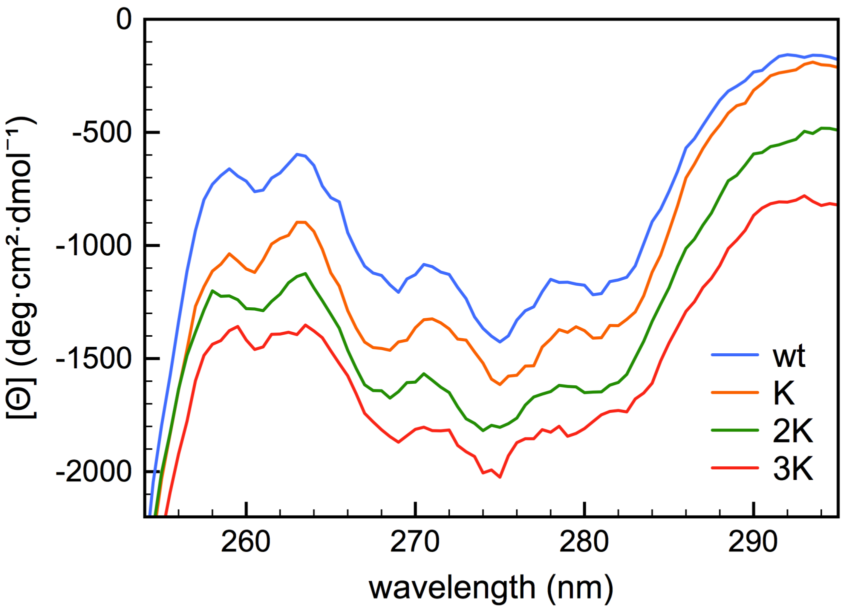

FIGURE S1. Near-UV CD spectra of 90 μM wt, K, 2K and 3K αSyn in 10 mM NH_4Ac, pH 7.40, measured at 25ºC. Spectra are arbitrarily offset for easier comparison. No clear differences among the E46K-like mutants are observed.

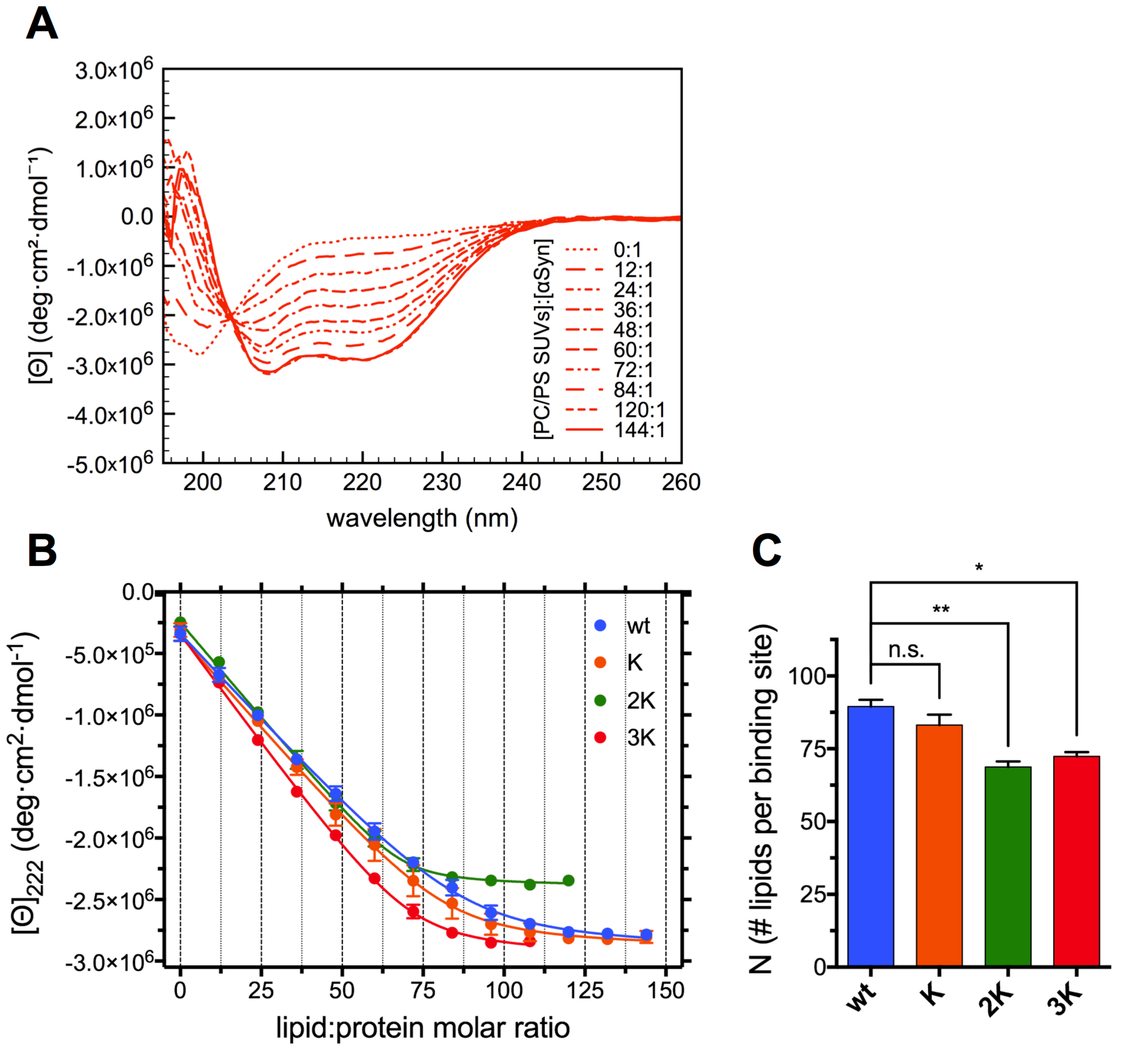

FIGURE S2. *A*, Representative far-UV CD spectra from a titration of 9.7 µM wt αSyn (in 9.7 mM NH_4Ac, 150 mM NaCl, pH 7.40) with 5.8 mM 70:30 POPC:DOPS SUVs, measured at 25ºC. *B*, Titration curves obtained from raw CD data after extracting the molar ellipticity at 222 nm and graphing it against the lipid:protein molar ratio at each point of the titration. Datapoints are shown along with their SEM (from *n*=3-6 independent titrations) and their best fit with an *N* independent binding sites model (*see* Experimental Procedures). *C*, Histogram of the *N* values (along with their SEM, *n*=3-6) obtained from the best fit of the titration curves with an *N* independent binding sites model. Decreasing *N*s indicate increased binding (or "avidity", *see* Results).

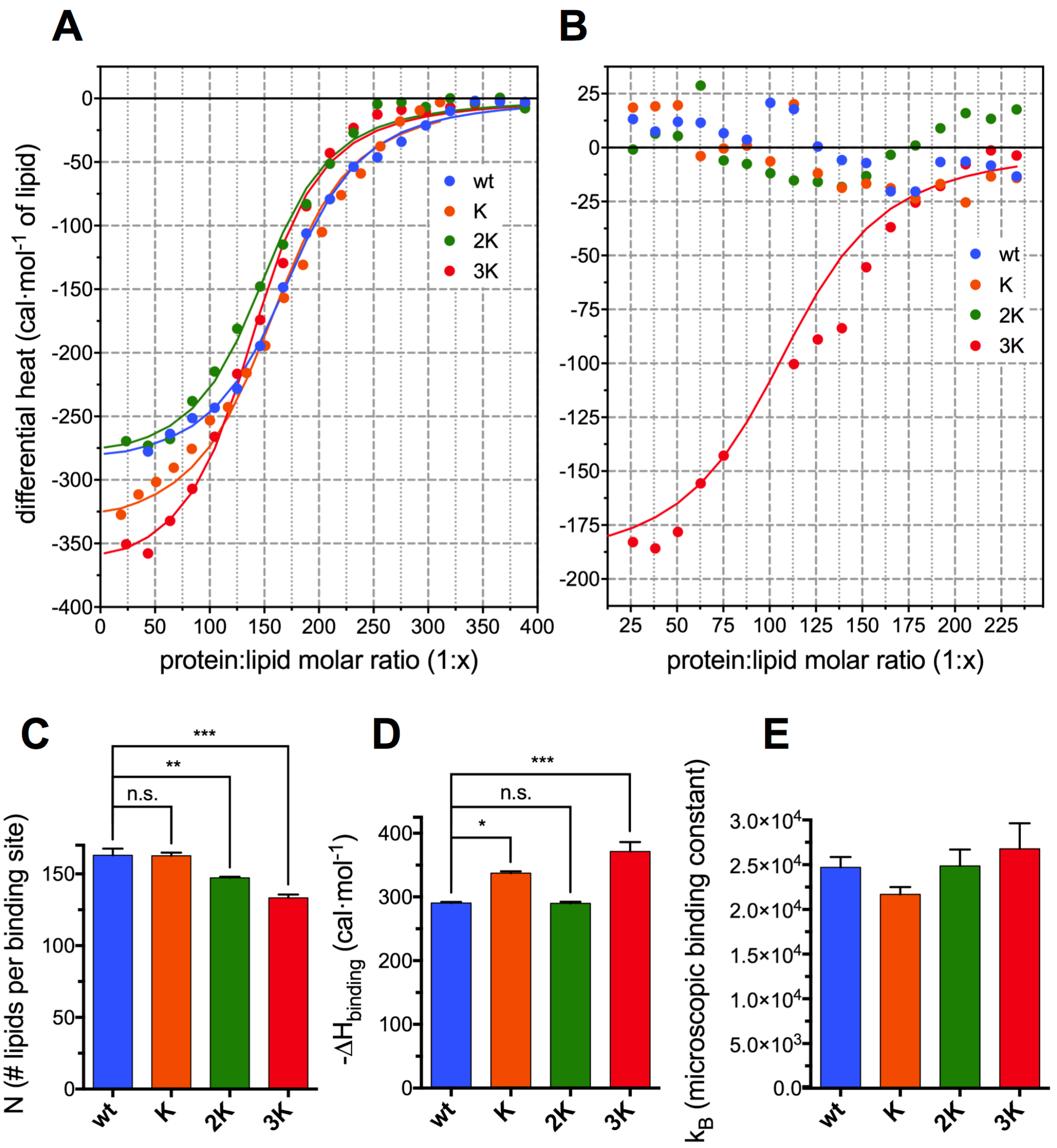

FIGURE S3. *A*, Representative ITC binding isotherms of 5 µM wt, K, 2K, and 3K αSyn (in 10 mM NH_4Ac, pH 7.40) with 6 mM 70:30 POPC:DOPS SUVs, measured at 25ºC. Datapoints are shown along with their best fit with an *N* independent binding sites model (*see* Experimental Procedures and Table S3). *B*, Representative ITC binding isotherms of 5 µM wt, K, 2K, and 3K αSyn (in 10 mM NH_4Ac, pH 7.40) with 6 mM 52.5:17.5:30 POPC:DOPS:cholesterol SUVs, measured at 25ºC. 3K αSyn datapoints are shown along with their best fit with an *N* independent binding sites model (*see* Experimental Procedures and Table S3). *C*, Histogram of the *N* values (along with their SEM, *n*=3) obtained from the best fit of the titration curves with an *N* independent binding sites model. Decreasing *N*s indicate increased binding (or "avidity", *see* Results). *D*, Histogram of the ΔH ($-\Delta H$) values (along with their SEM, *n*=3) obtained from the best fit of the titration curves with an *N* independent binding sites model. *E*, Histogram of the k_B values (along with their SEM, *n*=3) obtained from the best fit of the titration curves with an *N* independent binding sites model.

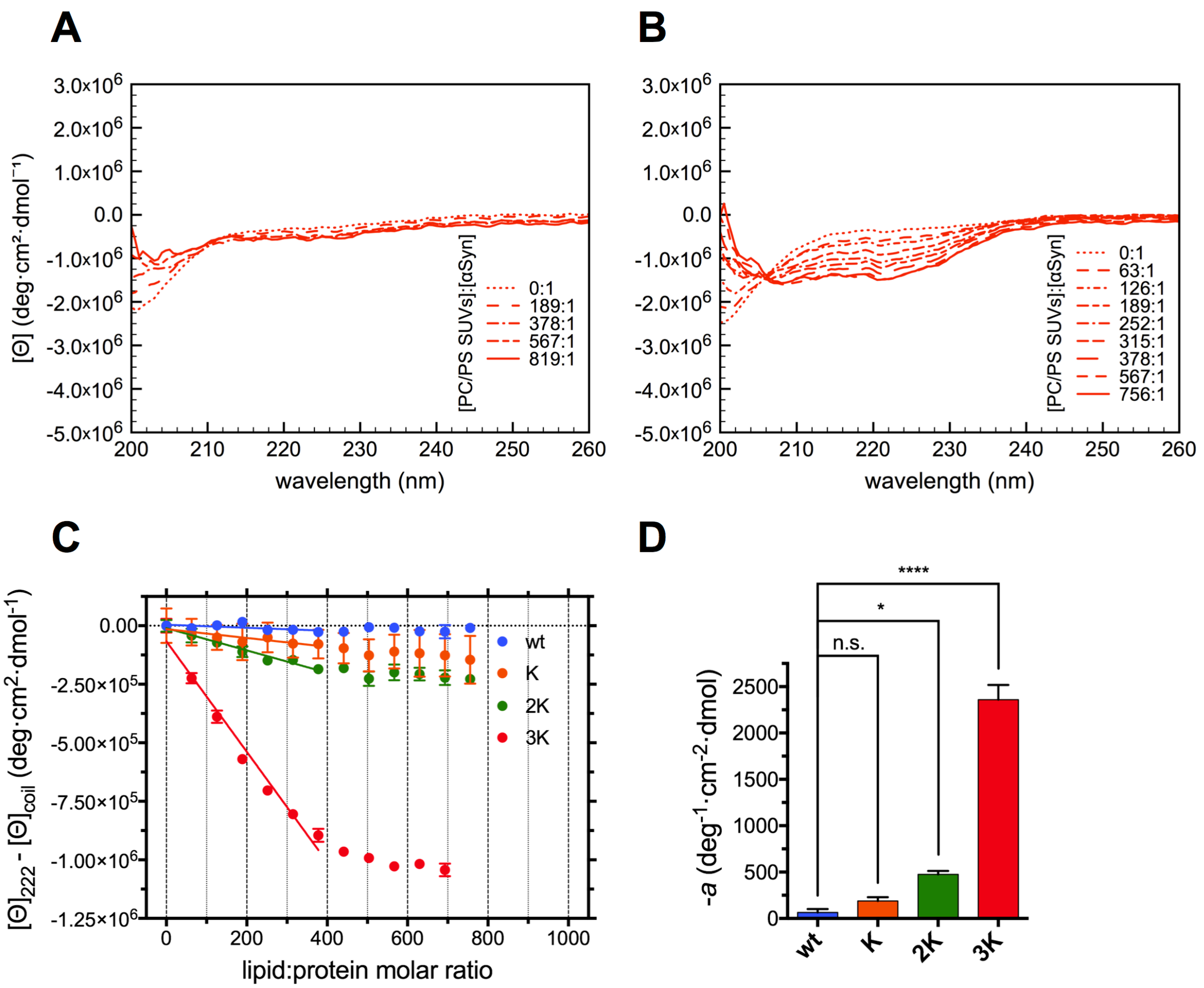

FIGURE S4. Representative far-UV CD spectra from a titration of 9.7 μM wt (*A*) and 3K (*B*) αSyn (in 9.7 mM NH_4Ac, 150 mM NaCl, pH 7.40) with 9.7 mM 70:30 POPC:DOPS LUVs, measured at 25ºC. No binding is observed in "high-salt" conditions with wt αSyn. *C*, Titration curves obtained from raw CD data after extracting the molar ellipticity at 222 nm, subtracting the unfolded ellipticity signal at the same wavelength and graphing it against the lipid:protein molar ratio at each point of the titration. Datapoints are shown along with their SEM (from *n*=3 independent titrations) and the best linear regression of the first 7 datapoints ($[\Theta]_{222} - [\Theta]_{coil} = a\cdot$(lipid:protein molar ratio)$+b$). *D*, Histogram of the slopes -*a* (along with their SEM, *n*=3) obtained from the linear regression of the titration curves. Increasing slopes (-*a*) indicate increased binding propensity.

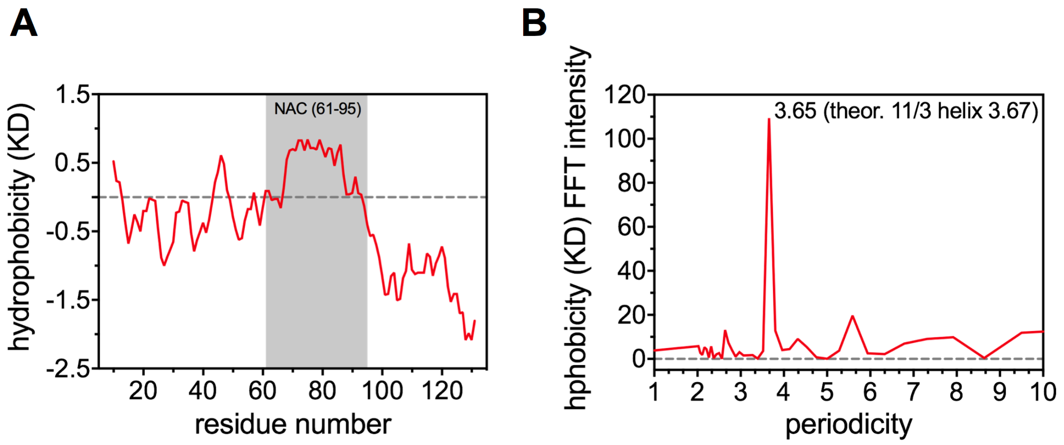

FIGURE S5. *A*, Hydropathy plot of the primary sequence of wt αSyn (1-140), calculated using the hydropathy index values of Kyte and Doolittle and averaged over a 21-residue moving window. The hydrophobicity of the primary sequence of αSyn highlights its key features: the amphipathic N-terminal region (1-95), with the slightly hydrophobic NAC domain (61-95, highlighted in gray), and the polar, negatively charged C-terminus (96-140). *B*, Fast Fourier Transform (FFT) analysis of the hydrophobicity of the N-terminus (1-95) of wt αSyn (again, the hydropathy index values of Kyte and Doolittle were used). The FFT shows a sharp maximum corresponding to the periodicity of an 11/3 helix (measured as number of residues per helix turn).

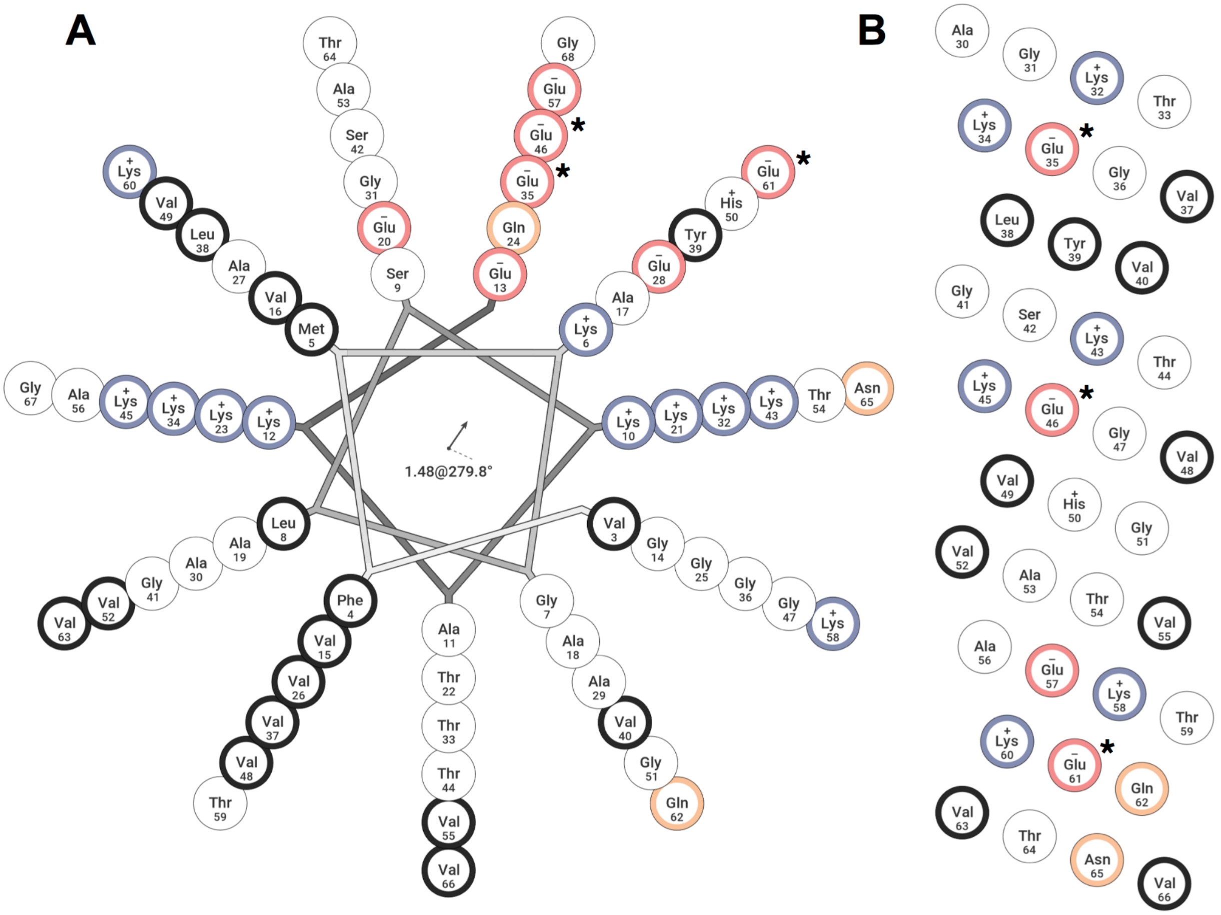

FIGURE S6. *A*, Schiffer-Edmundson helical wheel diagram of wt αSyn's N-terminal region (3-68), with the amino acids color-coded according to their polarity (Blue: positively charged aa, Red: negatively charged aa, Yellow: polar aa, White: neutral aa, Black: apolar aa). E61 is shown to be slightly closer to the membrane surface and thus comparatively better positioned to establish ionic bonds with the negatively charged lipid heads upon E-to-K mutation. *B*, Helical net diagram of the segment 30-66 of wt αSyn, color-coded as in *A*. In addition to the helical orientation, the local environment of E61 is also different from E35 and E46, suggesting that the rearrangement of electrostatic and/or hydrogen bonds upon E-to-K mutation could explain an increased 11/3 helix stability and membrane avidity.

TABLE S1 (.xlsx). ^{1}H-^{15}N HSQC spectral parameters (assigned chemical shifts and chemical shift perturbations) for Figs. 2-3.

	**Hydrodynamic Radius (nm)**
POPC:DOPS SUVs (sonicated)	16.4±0.9
POPC:DOPS:cholesterol SUVs (sonicated)	17.8±0.1
POPC:DOPS LUVs (extruded, 0.1 μm)	48±2

TABLE S2. DLS-measured hydrodynamic radii (along with their SEM, *n*=3) of freshly-prepared 5 mM 70:30 POPC:DOPS SUVs, 52.5:17.5:30 POPC:DOPS:cholesterol SUVs and 70:30 POPC:DOPS LUVs (extruded through a 0.1-μm polycarbonate membrane), measured at 20ºC.

**CD**	*N*	k_B	$-[\Theta]^{222}_{helix}$ *(deg·cm²·dmol⁻¹)*
wt PC:PS SUVs	157±5	(7±4)·10^4	(3.59±0.07)·10^6
K PC:PS SUVs	151±4 (n.s.)	(8±4)·10^4 (n.s.)	(3.83±0.08)·10^6 (n.s.)
2K PC:PS SUVs	148±4 (n.s.)	(8±4)·10^4 (n.s.)	(3.74±0.05)·10^6 (n.s.)
3K PC:PS SUVs	128±3 (**)	(1.1±0.5)·10^4 (n.s.)	(3.5±0.1)·10^6 (n.s.)
wt PC:PS:chol SUVs	164±3	(8±2)·10^4	(3.15±0.05)·10^6
K PC:PS:chol SUVs	165±3 (n.s.)	(8±3)·10^4 (n.s.)	(3.73±0.04)·10^6 (**)
2K PC:PS:chol SUVs	167±3 (n.s.)	(6±2)·10^4 (n.s.)	(3.44±0.04)·10^6 (n.s.)
3K PC:PS:chol SUVs	133±3 (***)	(9±3)·10^4 (n.s.)	(3.2±0.2)·10^6 (n.s.)
wt PC:PS SUVs+NaCl	90±2	(4±2)·10^4	*N/A*
K PC:PS SUVs+NaCl	83±4 (n.s.)	(5±4)·10^4 (n.s.)	*N/A*
2K PC:PS SUVs+NaCl	69±2 (**)	(1.3±0.9)·10^5 (n.s.)	*N/A*
3K PC:PS SUVs+NaCl	72±2 (*)	(6±3)·10^4 (n.s.)	*N/A*
wt PC:PS LUVs	(8.2±0.2)·10^2	(1.0±0.2)·10^3	(2.54±0.06)·10^6
K PC:PS LUVs	(6.3±0.3)·10^2 (**)	(1.4±0.5)·10^3 (n.s.)	(2.65±0.09)·10^6 (n.s.)
2K PC:PS LUVs	(3.9±0.3)·10^2 (****)	(1.3±0.4)·10^3 (n.s.)	(2.85±0.07)·10^6 (*)
3K PC:PS LUVs	(2.6±0.2)·10^2 (****)	(3±1)·10^3 (n.s.)	(2.93±0.06)·10^6 (*)

**ITC**	*N*	k_B	*-ΔH (cal·mol⁻¹)*	*ΔS (cal·mol⁻¹·K⁻¹)*
wt PC:PS SUVs	163±5	(2.5±0.1)·10^4	290±2	19.1±0.1
K PC:PS SUVs	163±2 (n.s.)	(2.17±0.08)·10^4 (n.s.)	338±3 (*)	18.7±0.1 (n.s.)
2K PC:PS SUVs	147±1 (*)	(2.5±0.2)·10^4 (n.s.)	290±2 (n.s.)	19.1±0.2 (n.s.)
3K PC:PS SUVs	133±2 (***)	(2.7±0.3)·10^4 (n.s.)	(3.7±0.1)·10^2 (***)	19.0±0.3 (n.s.)
3K PC:PS:chol SUVs	95±8 (N/A)	(2.3±0.3)·10^4 (N/A)	(1.7±0.1)·10^2 (N/A)	19.3±0.3 (N/A)

**CLEARANCE KINETICS**	$t_{1/2}^{slow\,(abs)}$ (s^{-1})	$t_{1/2}^{fast\,(abs)}$ (s^{-1})	$t_{1/2}^{slow\,(CD)}$ (s^{-1})	$t_{1/2}^{fast\,(CD)}$ (s^{-1})
wt PC:PG MLVs	(818.3, 939.7)	(88.5, 102.2)	(926.0, 1046.0)	(125.6, 145.9)
K PC:PG MLVs	(712.3, 806.5)	(61.7, 70.6)	(615.2, 666.7)	(72.2, 84.9)
2K PC:PG MLVs	(305.3, 458.8)	(79.5, 113.3)	(300.9, 356.3)	(38.4, 64.8)
3K PC:PG MLVs	(289.4, 343.9)	(23.6, 35.6)	(419.1, 523.9)	(46.8, 67.2)

TABLE S3. Summary table of the best-fit model parameters. CD-monitored (222-nm ellipticity) titrations of wt, K, 2K, and 3K αSyn with 70:30 POPC:DOPS SUVs, 52.5:17.5:30 POPC:DOPS:cholesterol SUVs, 70:30 POPC:DOPS SUVs in 150 mM NaCl, and 70:30 POPC:DOPS LUVs; best fits obtained with an *N* independent binding sites model (*n*=3, SEMs are shown). ITC binding isotherms of wt, K, 2K, and 3K

αSyn with 70:30 POPC:DOPS SUVs and 3K αSyn with 52.5:17.5:30 POPC:DOPS:cholesterol SUVs; best fits obtained with an *N* independent binding sites model (*n*=3, SEMs are shown). Vesicle clearance kinetics of 90:10 POPC:POPG MLV dispersions by wt, K, 2K, and 3K αSyn, monitored both by absorbance at 500 nm and by CD (222-nm ellipticity); best fits obtained with a two-phase exponential decay model (95% CIs are shown). Statistical significance was determined by ordinary one-way ANOVA followed by Sidak's multiple comparisons test (vs. wt αSyn), with a single pooled variance; n.s. $p>0.05$, * $p\leq0.05$, ** $p\leq0.01$, *** $p\leq0.001$, **** $p\leq0.0001$.
